# Beyond the Usual: A Rare Case of Tracheoesophageal Fistula

**DOI:** 10.7759/cureus.93288

**Published:** 2025-09-26

**Authors:** Victoria Maldonado, Gabriela Guadalupe Rios, Robert Dorman, Sreekanth K Viswanathan, Caroline Chua

**Affiliations:** 1 Division of Neonatology, Nemours Children's Health, Orlando, USA; 2 Department of Surgery, Nemours Children's Health, Orlando, USA

**Keywords:** congenital abnormality, esophageal atresia, infant, secondary fistula, tracheoesophageal fistula

## Abstract

Esophageal atresia (EA) with tracheoesophageal fistula (TEF) is an uncommon congenital abnormality, and double TEF is observed in less than 1% of all EA/TEF cases. Additionally, recurrence of EA with TEF in the offspring of affected individuals is also considered a rare condition.

We present the case of an early-term infant with a paternal family history of EA/TEF who was diagnosed with EA and a distal TEF (type C EA/TEF), underwent primary repair and ligation, and was subsequently found to have a second proximal fistula (type D TEF). This case highlights the diagnostic challenges associated with double TEF and emphasizes the importance of thorough evaluation in all EA/TEF presentations. Maintaining a high index of suspicion is essential to ensure all anatomical variants are identified and appropriately managed. Familial recurrence of isolated EA/TEF, while infrequent, should also be considered in clinical assessment and counseling.

## Introduction

Esophageal atresia (EA) with tracheoesophageal fistula (TEF) is a rare congenital anomaly with an incidence of about 1 in 2500 to 4000 live births [1-4]. Esophageal atresia can be isolated, but it usually occurs with TEF. The Gross classification system is the most widely used method for categorizing EA and TEF based on anatomical features. The most common type is Gross type C, which consists of EA with a distal TEF, accounting for approximately 85% of cases; Type D, characterized by both proximal and distal fistulas, is much less frequently encountered [5-8]. Infants born with EA/TEF do not always present symptoms at birth. The anomaly is typically discovered when a patient has copious secretions, respiratory distress, and failure to pass an orogastric or nasogastric tube. In utero, polyhydramnios or a dilated esophageal pouch may suggest EA/TEF.

The etiology of EA/TEF is not fully understood. Current literature offers several theories, including a genetic component. However, isolated EA/TEF has been reported to recur in offspring with a frequency of approximately 1-3% [1,5,6]. Embryologically, it is hypothesized that the origin of EA with or without TEF occurs around days 25-37 of conception, during the separation of the foregut endoderm into the trachea and esophagus [6,9,10]. Several transcription factors (e.g., SOX2, FOXA2), molecular pathways (e.g., sonic hedgehog, Hox, retinoic acid), and growth factors (e.g., FGF4) may be related to the development of TEF [6,7]. Chromosomal aneuploidies, microdeletion 22q11.2, and other single-gene disorders (e.g., CHD7, SOX2, FANCB, MYCN, TCF4, NRXN) are reportedly associated with the development of TEF [6,7,10]. Other studies report a close association of fetal exposure with diethylstilbestrol, alcohol, cigarettes, or methimazole exposure, and maternal diabetes [2,7]. Overall, it is understood that EA with TEF appears to be multifactorial in etiology, including environmental exposures, genetics, and chromosomal anomalies.

Tracheoesophageal fistulas are more likely to occur with other congenital anomalies rather than in isolation. Two commonly seen associations include VACTERL (vertebral, anorectal, cardiac, tracheoesophageal, renal, and limb anomalies) and CHARGE (coloboma, heart defects, choanal atresia, restricted growth, genitourinary abnormalities, and ear abnormalities/deafness) [1,5-7,10]. Patients who present with EA/TEF require further evaluation for other associated anomalies, including, but not limited to, limb, genitourinary, renal, cardiac, and vertebral involvement. When EA/TEF is suspected or confirmed, patients usually undergo additional studies, such as radiographs, to assess structural abnormalities, echocardiograms, and renal ultrasound. Chromosomal abnormalities, particularly trisomy 21, have been associated with the presence of a TEF.

Surgical correction of EA/TEF has drastically decreased mortality [5]. Despite surgical correction, there are still various morbidities to monitor. Even with a successful repair, as patients grow into adulthood, they are at risk for complications such as stricture, secondary TEF formation, respiratory disease, recurrent respiratory infections, gastrointestinal disease, and malignancy of the esophagus [5,11].

## Case presentation

We present a male neonate with a birth weight of 2892 grams. He was born via spontaneous vaginal delivery at 37 weeks and 4 days of gestation, with no birth-related complications reported. Maternal prenatal labs and courses were unremarkable. In addition, there was no history of polyhydramnios. Apgar scores were 8 and 9 at 1 and 5 minutes, respectively. Shortly after birth, he developed respiratory distress with copious oral secretions. An orogastric tube could not be passed and was coiled in the esophagus on chest radiograph. Esophageal atresia was suspected, and the child was subsequently transferred to our institution for a higher level of care. Notably, the patient’s father had a personal history of TEF, which was successfully repaired during infancy without complications. Genetic testing was offered, but declined.

**Figure 1:**
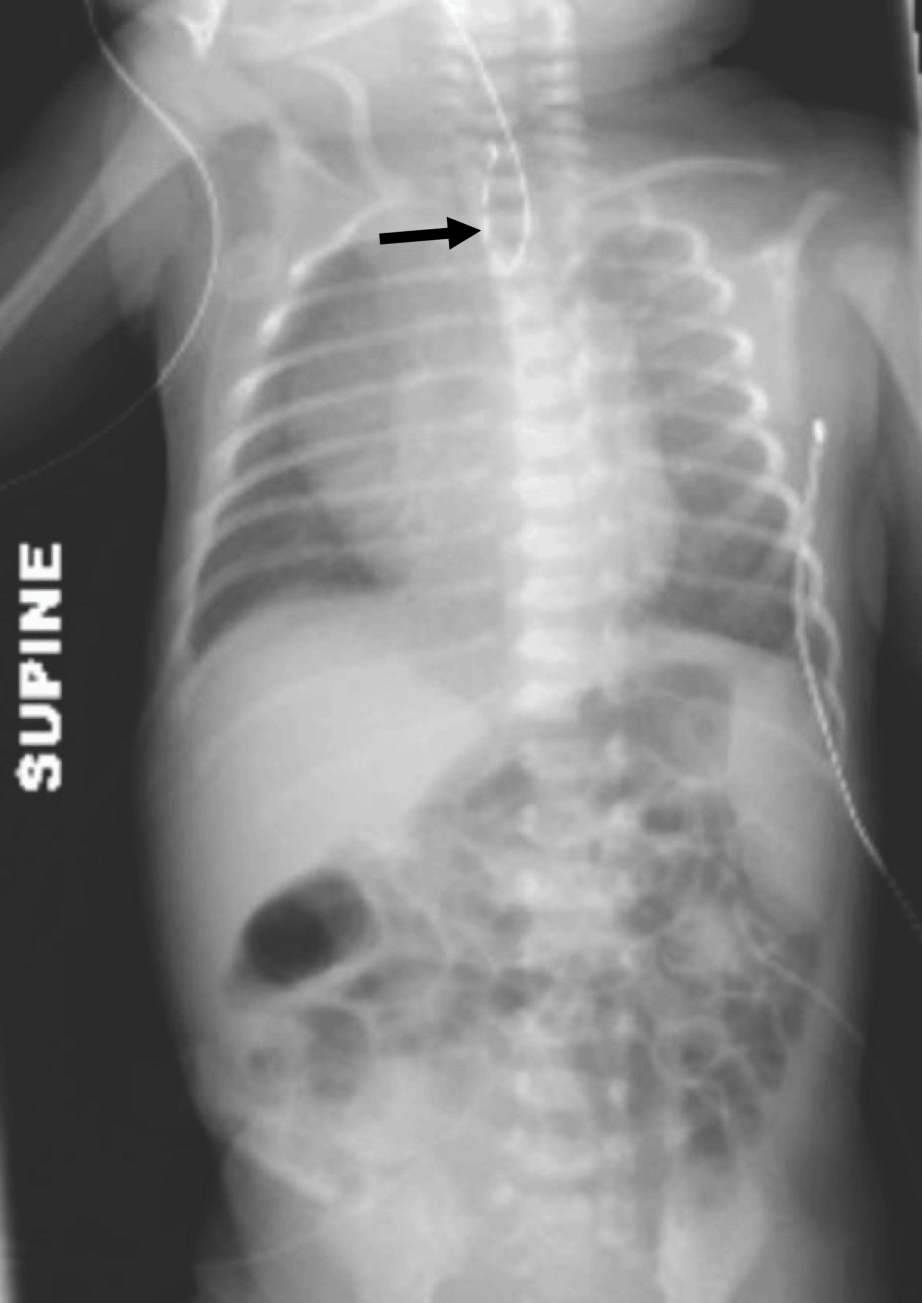
X-ray showing coiling of the nasogastric tube in the upper esophagus (black arrow) with bowel gas pattern consistent with tracheoesophageal fistula

Repeat radiographs showed the presence of intestinal air and the orogastric tube terminating in the thorax, which was consistent with EA/TEF (Figure 1). A VACTERL workup was initiated to look for other commonly associated anomalies. His echocardiogram, renal ultrasound, and vertebral radiographs were normal. Physical exam revealed a patent anus and no limb anomalies. The patient, however, was microcephalic with a head circumference below the third percentile. Urine polymerase chain reaction testing for cytomegalovirus in the presence of microcephaly was negative. Weight and length were appropriate for gestational age.

**Figure 2:**
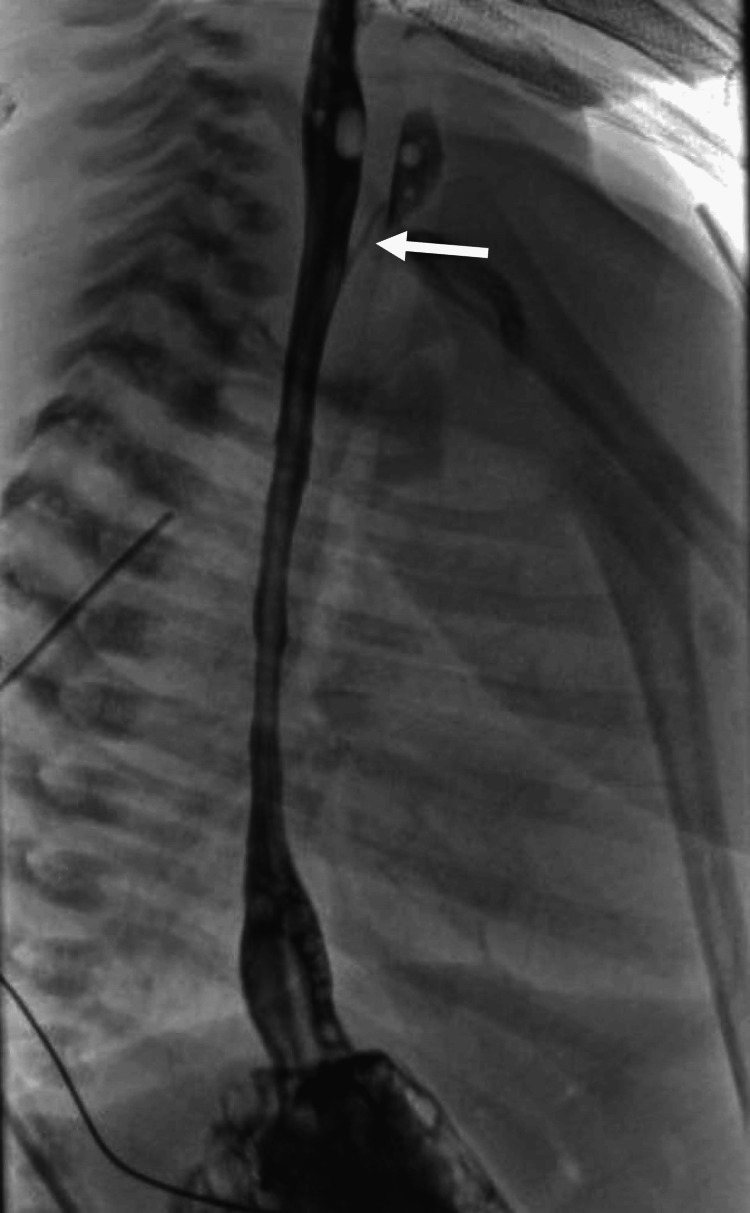
Fluoroscopy of the esophagus with contrast showing a type D tracheoesophageal fistula (white arrow).

The patient underwent surgical repair for type C EA/TEF on the second day of life (DOL). Laryngotracheoscopy was performed to assess for proximal fistula, and none was visualized. The patient was intubated for surgery, and primary repair was performed through a right thoracotomy utilizing an extra-pleural approach. Intraoperatively, the proximal and distal esophageal segments overlapped by approximately 1 cm, allowing for a tension-free, end-to-end primary anastomosis using interrupted absorbable sutures with only limited mobilization required; this was confirmed visually and by gentle approximation of the esophageal ends. A trans-anastomotic nasogastric tube was left in place for five days postoperatively, in accordance with institutional guidelines. A right-sided extra-pleural drain and trans-anastomotic nasogastric tube were left in place. The patient was extubated to high-flow nasal cannula the following day and then to unassisted room air on postoperative day (POD) 3. His feeds were slowly advanced from small-volume continuous feeds beginning on POD 4. Postoperatively, analgesia and sedation were managed with a combination of acetaminophen, fentanyl, and dexmedetomidine. A routine postoperative contrast swallow study on POD 5 revealed a patent anastomosis without leak, as well as a separate proximal pouch fistula around the C6-T1 region (Figure 2). The patient’s clinical course was further complicated by a new onset of respiratory distress requiring reinstitution of high-flow nasal cannula, concerning for developing infection. Peripheral blood cultures grew Klebsiella aerogenes; however, the cerebrospinal fluid studies were unremarkable. Based on the results of microbial sensitivity testing, initial empiric treatment with gentamicin and nafcillin was switched to cefepime for 10 days to treat the Klebsiella bacteremia. The patient's condition subsequently improved, allowing for de-escalation back to unassisted room air.

Surgical correction of the proximal TEF was deferred to allow the patient to recover from initial surgery. He was discharged home on DOL 27 with nasogastric feeding of expressed breast milk and daily vitamin D.

The patient was readmitted on DOL 51 for the scheduled second TEF repair surgery. Bronchoscopy confirmed the visualization of the previously repaired distal segment and the proximal portion of the type D TEF fistula prior to the surgical intervention. The proximal portion of the type D TEF was repaired by the general surgery and otolaryngology teams through a right cervical approach with active monitoring of the recurrent laryngeal nerve without intraoperative complications. On POD 4, a barium swallow study showed a patent esophagus with no evidence of recurrent or residual TEF at either of the repair sites. However, the patient continued to exhibit signs of tracheal aspiration with thin liquids, which necessitated continuing enteral feeds via a nasogastric tube. A repeat barium swallow study on POD 7 indicated that the patient was tolerating mildly thickened liquids orally. He was discharged home on POD 10 on full oral feeds with mildly thickened milk.

At the initial follow-up visit with the surgery team, nearly one month post-procedure, the patient was doing well and tolerating thickened feeds orally. His parents expressed concern about his noisy breathing, a symptom consistent with tracheomalacia, which is a known associated pathology in patients with a history of TEF.

## Discussion

The incidence of EA/TEF is low, and double TEF represents less than 1% of all EA/TEF cases [8,12]. Our case illustrates the difficulty in diagnosing a double TEF and highlights the importance of thorough preoperative and intraoperative evaluation to detect any additional anomalies and prevent postoperative complications. A 2013 case study reported a similar scenario in which a secondary, more proximal fistula was discovered on postoperative barium esophagogram (as seen in our patient) [8]. Another case study highlighted the presence of a double TEF accompanied by an even rarer phenomenon, the absence of EA [13]. In this case as well, a more proximal fistula was discovered postoperatively. Fistulas are often difficult to locate if they are small, as the tracheal mucosa often collapses on itself, thus obscuring the fistula [14].

A 2005 review discussed 23 globally reported cases of EA with double TEF [15]. In all cases, a distal TEF was diagnosed preoperatively, while in five cases, a second, more proximal fistula was identified preoperatively [15]. About one-third of the cases reported the discovery of a second, more proximal fistula during the intraoperative TEF ligation procedure. The remaining cases were diagnosed postoperatively, and these patients had associated complications such as frequent pulmonary infections [15]. Although secondary fistulas are infrequent, there have been well-documented cases that reported discovery either intraoperatively or postoperatively [14,15]. Regrettably, secondary fistulas were detected only post-mortem in a few cases, with presumed causes of death related to pulmonary complications [15].

When patients remain symptomatic post-repair, the presence of an undetected secondary fistula should be pursued. However, the development of a fistula is a known complication of the repair procedure itself. It is crucial to differentiate between a missed secondary fistula and a fistula resulting from the surgical intervention. Since the location of the proximal fistula was at the C6-T1 level, as demonstrated on the postoperative contrast study, this is most consistent with a missed congenital proximal fistula rather than a recurrent anastomotic fistula, which typically occurs adjacent to the anastomosis and presents later in the postoperative course [15].

Careful preoperative and intraoperative evaluation, including thorough an endoscopic assessment of the entire trachea and esophagus, is essential, as small proximal fistulas may be easily missed due to their size or location and the tendency of tracheal mucosa to obscure small defects, especially in the absence of active airflow or positive pressure [8]; postoperative imaging should be scrutinized for evidence of additional fistulas. Adjunctive diagnostic techniques, such as rigid bronchoscopy by pediatric airway specialists, selective dye instillation (e.g., methylene blue), or capnography/CO₂ detection in the esophagus, and careful ventilation maneuvers may improve detection rates in challenging cases [3,8].

Persistent or recurrent respiratory or feeding difficulties after EA/TEF repair should prompt re-evaluation for a missed secondary fistula. Early recognition and multidisciplinary management are critical to optimize outcomes and minimize complications.

The recurrence rate of EA/TEF in offspring is <2%, making our case extremely unique. Our patient had an isolated TEF just like his father; however, the type of TEF in his father was unknown. Our patient had a type D TEF.

## Conclusions

This case highlights the diagnostic challenges associated with double TEF, a rare variant of EA that accounts for less than 1% of all EA/TEF cases. Our patient, with a paternal history of EA/TEF, presented with type C EA/TEF and was later found to have an additional proximal fistula (type D EA/TEF) on postoperative imaging. This emphasizes the importance of maintaining a high index of suspicion for multiple fistulas, particularly when postoperative symptoms persist or recur. This case also illustrates the rare familial recurrence of EA/TEF, supporting the need for genetic counseling and ongoing follow-up in affected families.
